# Supplementary material for: Mitochondrial complex III subunit Qcr8 regulates the virulence and adhesion of Candida albicans by modulating mitochondrial function
Source: Microbiol Spectr. 2025 Dec 8;14(1):e01672-25. doi: 10.1128/spectrum.01672-25 (PMC12772293; doi:10.1128/spectrum.01672-25)
Supplement: Figures S1 to S10; Tables S1 and S2 — Additional experiments. [file spectrum.01672-25-s0001.docx]

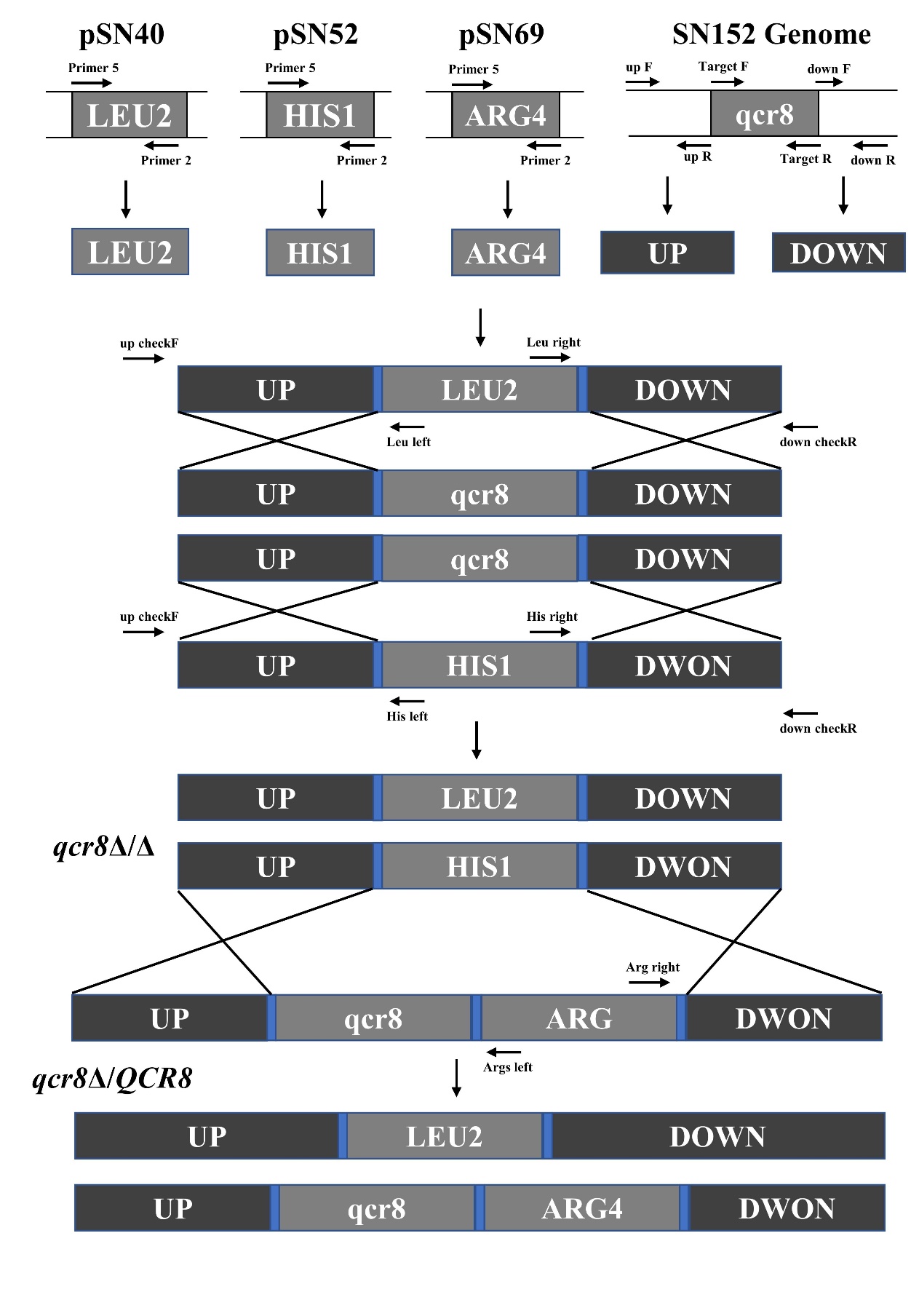
 **Figure S1. Schematic diagram of the *QCR8* knockout strategy using PCR-based homologous recombination.** The following primers were used in the strategy: upstream amplification primers (up F and up R); downstream amplification primers (down F and down R); universal amplification primers for the nutritional selection marker genes *LEU2*, *HIS1*, and *ARG4* (primer2 and primer5); and verification primers (up check F, down check R, Leu left, Leu right, His left, His right, target check F, and target check R).


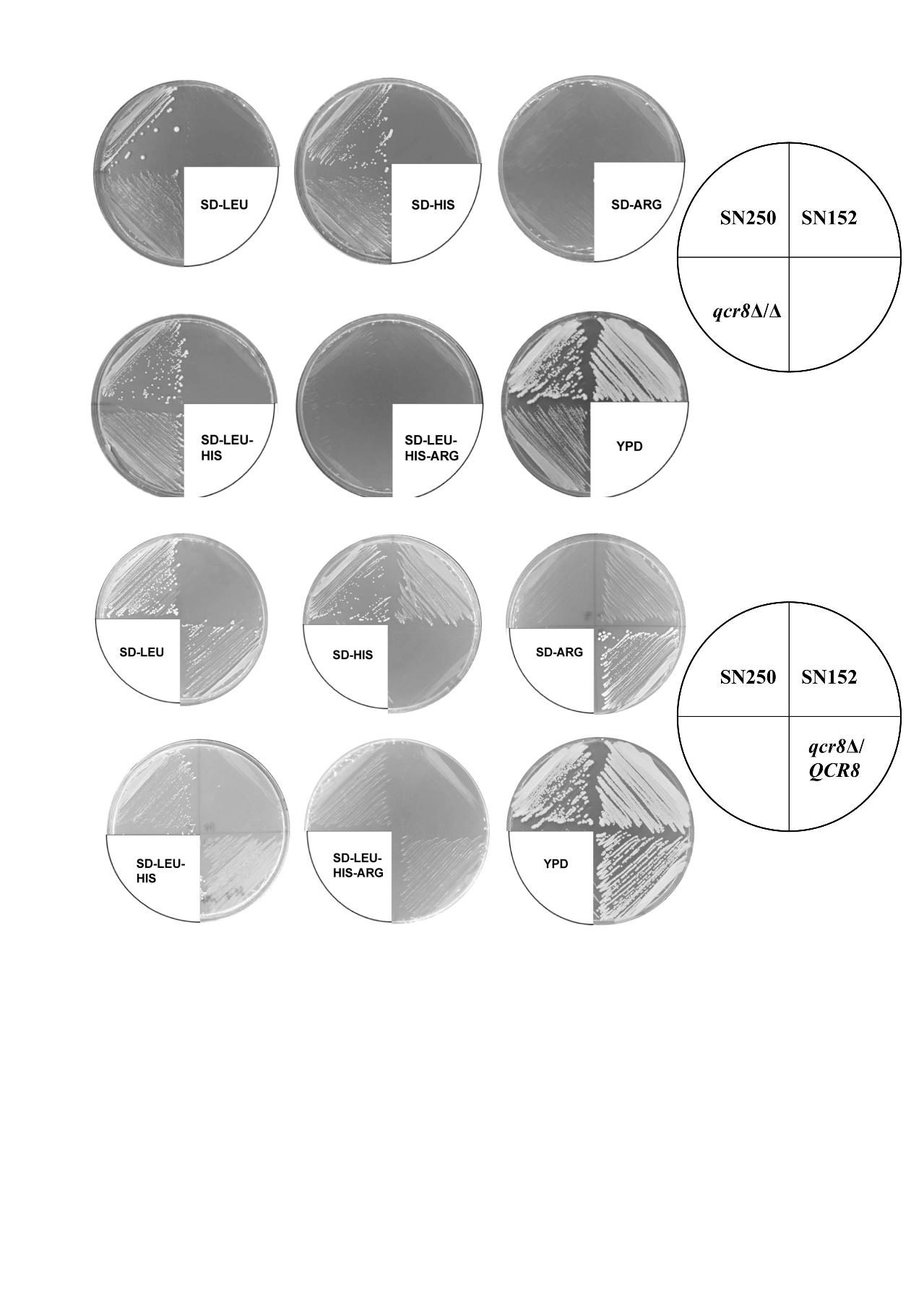


**Figure S2. Identification of *QCR8* mutant and *QCR8* complementary nutritional types.** SN152 **(***his1*Δ*/his1*Δ*, arg4*Δ*/arg4*Δ*, leu2*Δ*/leu2*Δ*, ura3*Δ*/URA3, iro1*Δ*/IRO1*) only grew on the YPD plate. SN250 (*leu2*Δ/*LEU2, his1*Δ/*HIS1, arg4*Δ*/arg4*Δ*, ura3*Δ*/URA3, iro1*Δ*/IRO1*) grew on the SD-LEU, SD-HIS, and YPD plates. The *QCR8* mutant grew on the SD-LEU, SD-HIS, and YPD plates. The *QCR8* complementary strain (*leu2*Δ/*LEU2*, *his1*Δ*/his1*Δ*, arg4*Δ*ARG4*Δ*, leu2*Δ*/leu2*Δ*, ura3*Δ*/URA3, iro1*Δ*/IRO1*) grew on the SD-LEU, SD-ARG, and YPD plates.


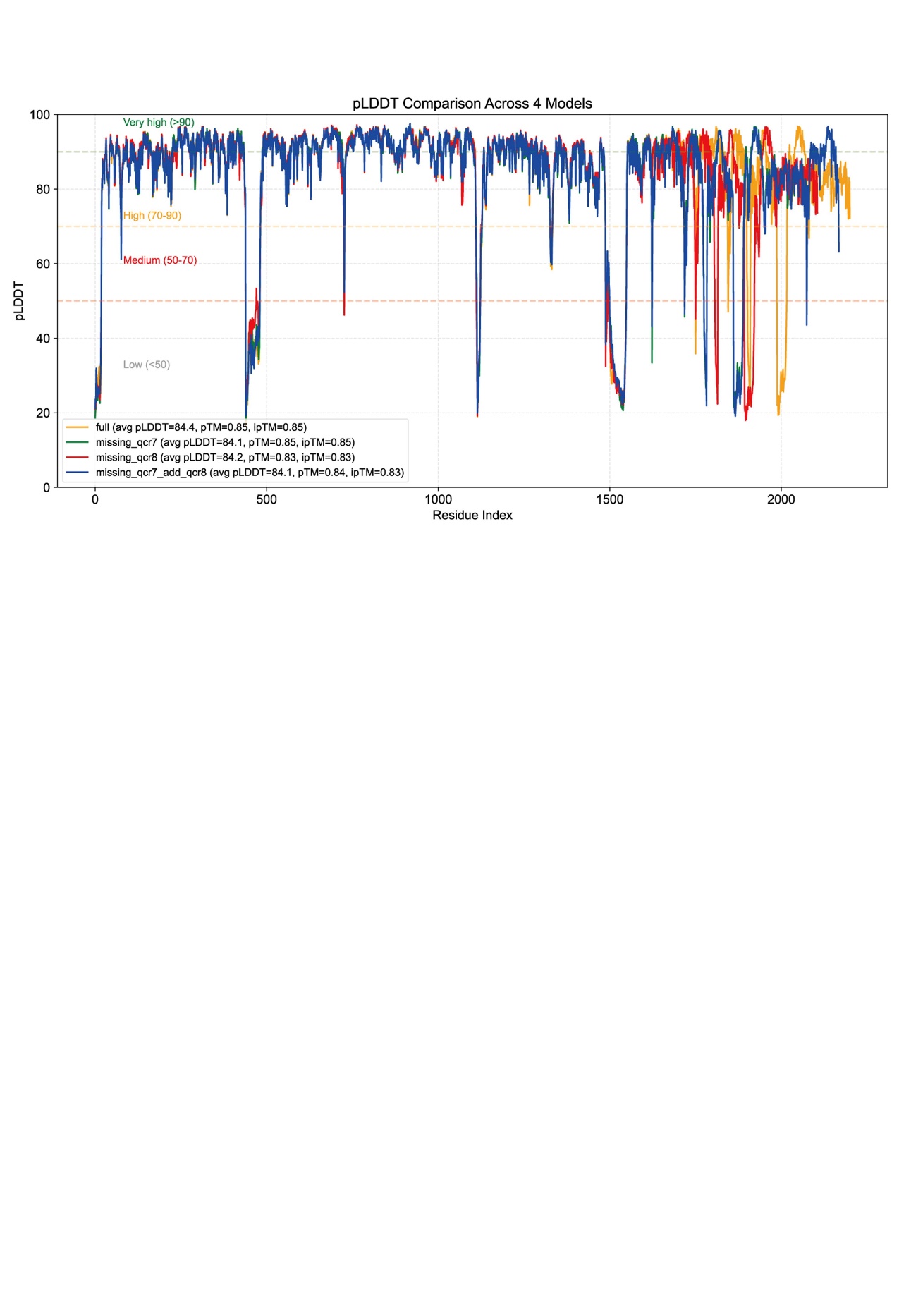


**Figure S3. Score predictions of four prediction models on the local distance difference test (pLDDT).** pLDDT reflects the prediction accuracy of each amino acid, and it ranges from 0 to 100. A value below 50 indicates a very inaccurate prediction or an intrinsically disordered protein; A value below 70 indicates low confidence; and a value above 90 indicates extremely high confidence.


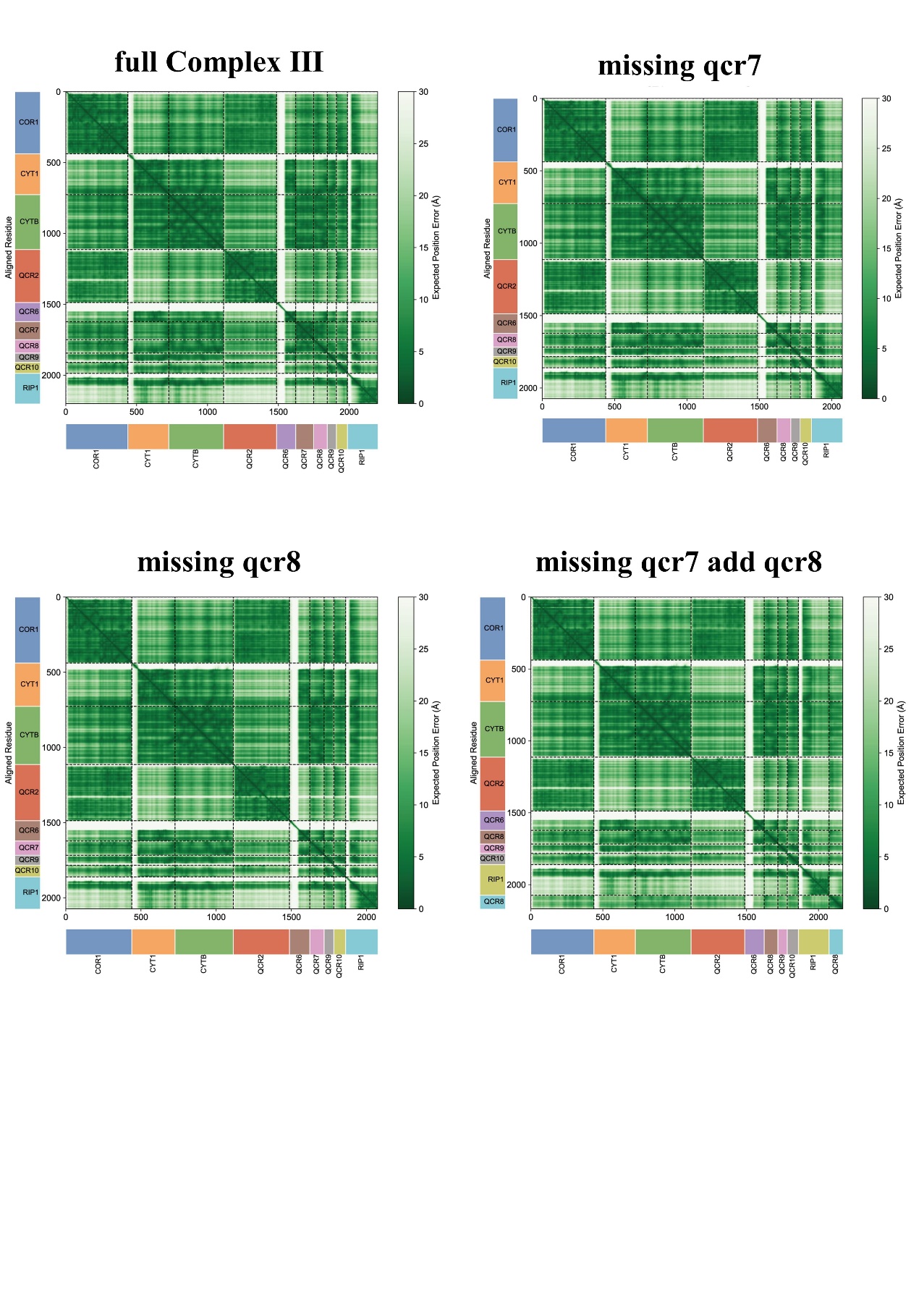


**Figure S4. PAE analysis of the four prediction models**. PAE represents the predicted difference between the distance of each pair of amino acids and the true value. The range is from 0 to 30, with lower values being better. The unit is angstroms (Å). In the figure, the greener the color, the better the performance.


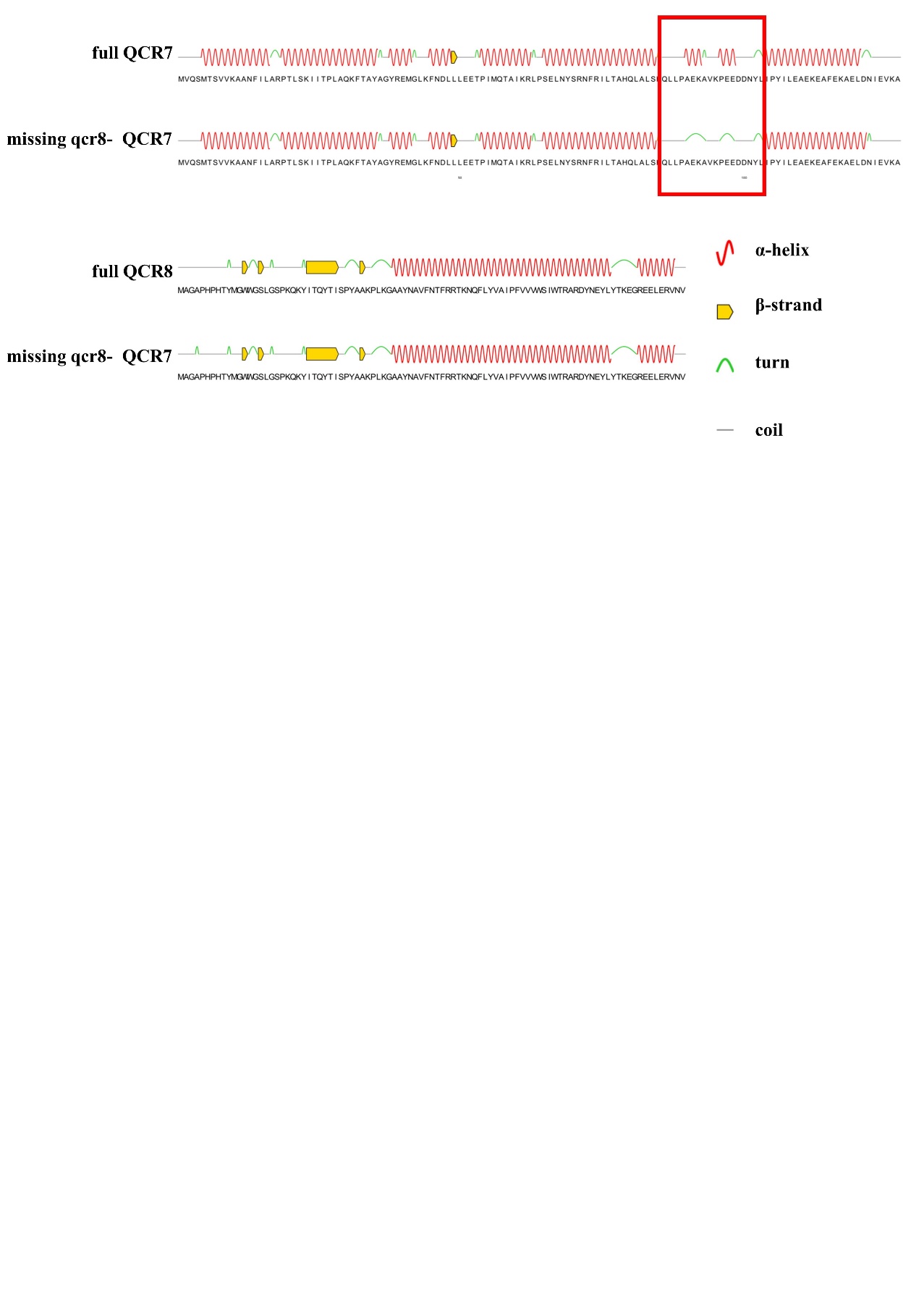


**Figure S5. DSSP analysis of Qcr7 and Qcr8.** The missing Qcr8 in the secondary structure diagram led to the loss of the Qcr7 α-helix region, while the missing Qcr7 had little effect on Qcr8, suggesting that the missing Qcr8 may alter the stability, activity, or interactions with other subunits of the complex.

**
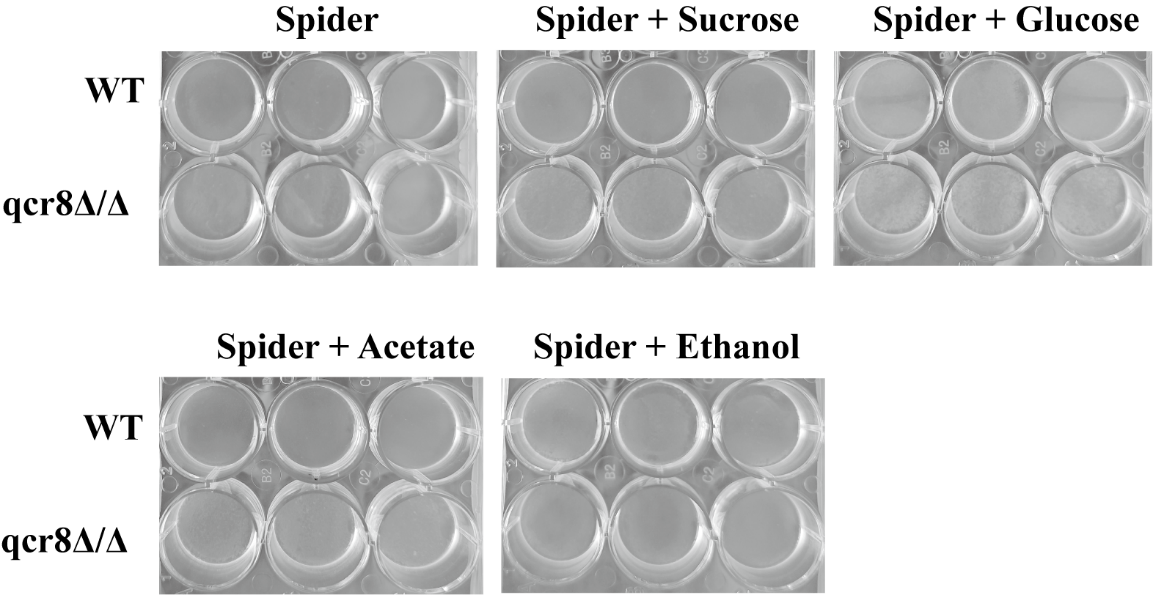
**

**Figure S6. Unwashed condition of the adhesion assay.** Under the unwashed condition, without using PBS to remove the non-adherent cells, no significant differences were observed between the WT and qcr8Δ/Δ strains.


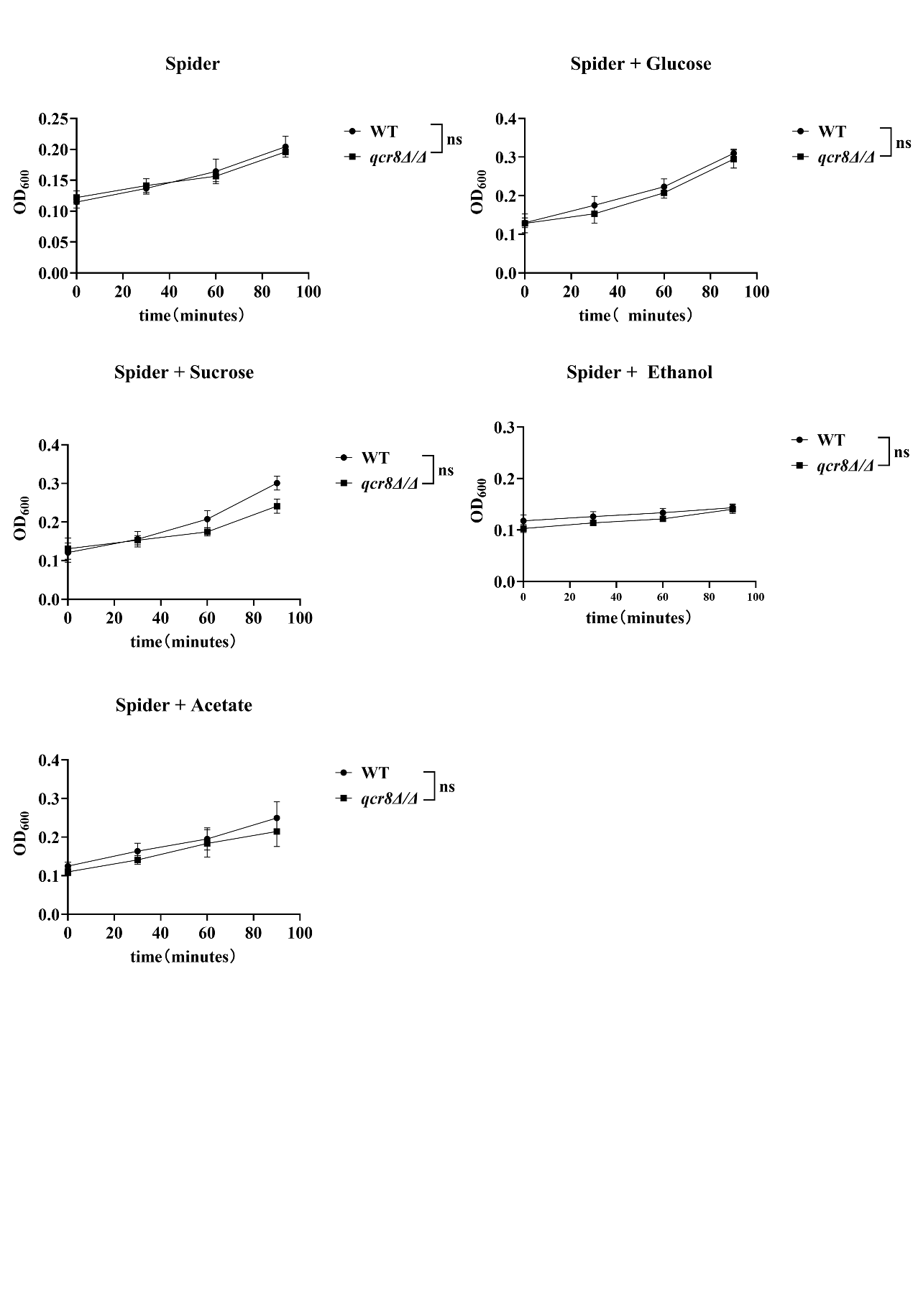


**Figure S7. Growth curves of WT and qcr8 under different carbon sources for 90 minutes.** After replacing the original carbon source (mannose) in Spider medium with glucose, sucrose, ethanol, or acetate, no significant differences in growth rates were observed between WT and qcr8 in Spider, Spider + Glucose, Spider + Sucrose, Spider + Ethanol, or Spider + Acetate.

*
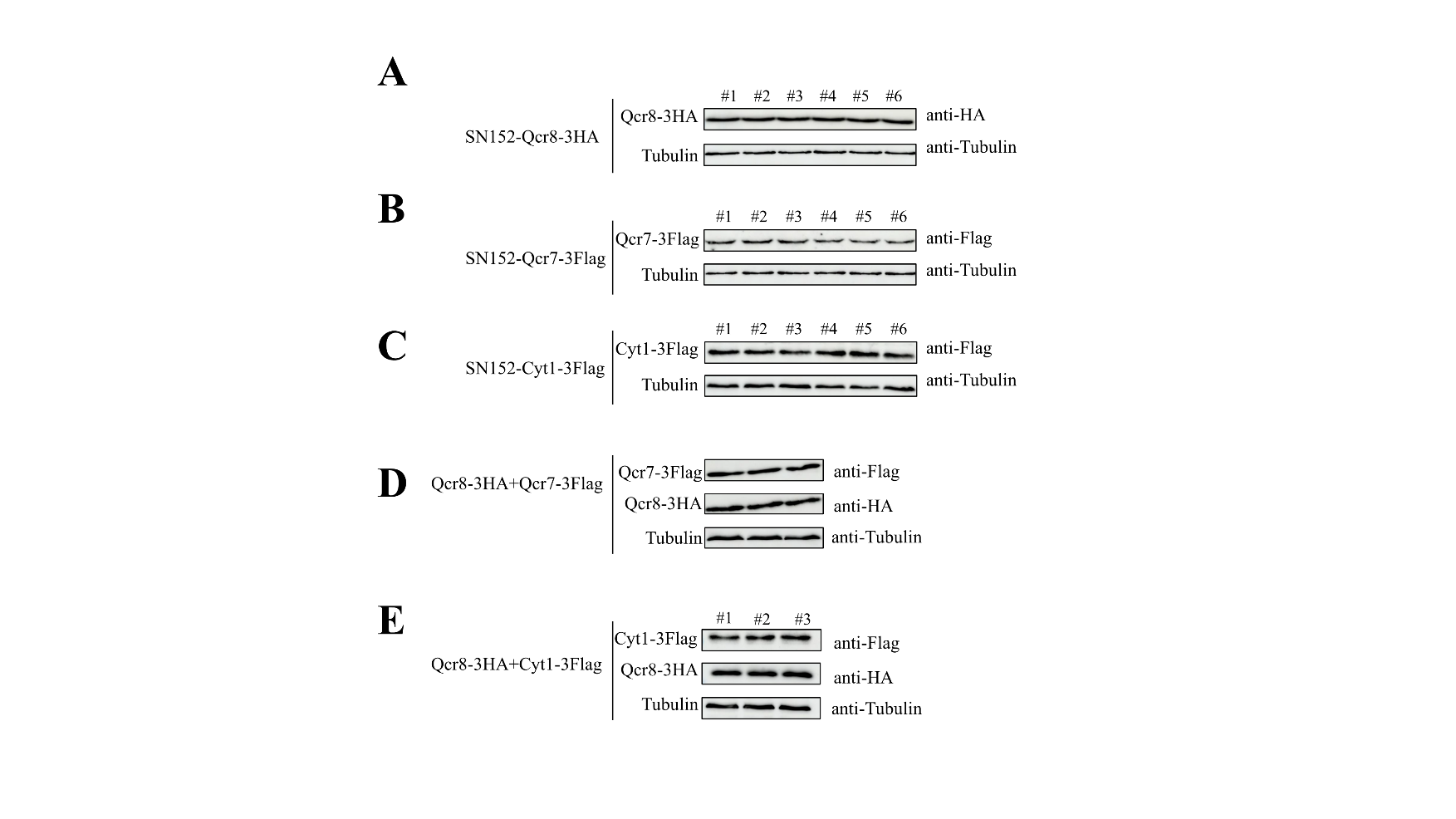
*

**Figure S8 Western blot verification of tagged strains constructed in different strain backgrounds.**(A) Construction of the Qcr8-3HA strain in the SN152 background; six positive clones were selected and verified by Western blot analysis.
(B) Construction of the Qcr7-3Flag strain in the SN152 background; six positive clones were selected and verified by Western blot analysis.
(C) Construction of the Cyt1-3Flag strain in the SN152 background; six positive clones were selected and verified by Western blot analysis.
(D) Construction of the Qcr7-3Flag strain in the Qcr8-3HA background; three positive clones were selected and verified by Western blot analysis.
(E) Construction of the Cyt1-3Flag strain in the Qcr8-3HA background; three positive clones were selected and verified by Western blot analysis.

**
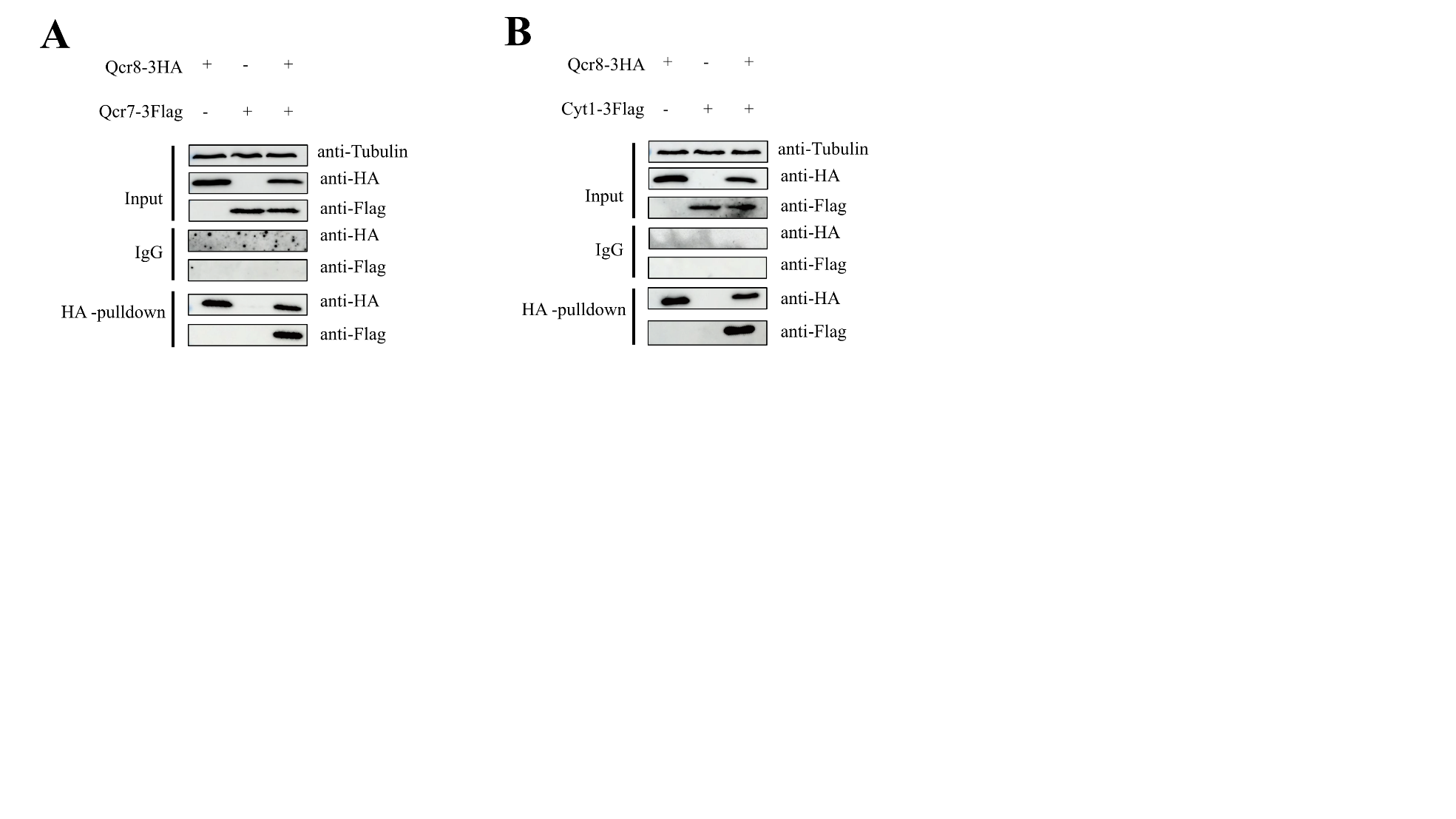
**

**Figure S9 Co-immunoprecipitation analysis of the interaction between Qcr8 and Qcr7 or Cyt1.**Input: total protein lysate without immunoprecipitation using anti-HA beads.
IgG: proteins immunoprecipitated using anti-IgG beads as a negative control.

(A) HA-tagged Qcr8 and Flag-tagged Qcr7 were co-expressed in the SN152 background. Cell lysates were immunoprecipitated with anti-HA beads and analyzed by Western blotting with the indicated antibodies. Anti-Tubulin served as a loading control. Detection of Qcr7-Flag in the HA-pulldown fraction indicates an interaction between Qcr8 and Qcr7.

(B) HA-tagged Qcr8 and Flag-tagged Cyt1 were co-expressed in the SN152 background. Cell lysates were immunoprecipitated with anti-HA beads and analyzed by Western blotting with the indicated antibodies. Anti-Tubulin served as a loading control. Detection of Cyt1-Flag in the HA-pulldown fraction indicates an interaction between Qcr8 and Cyt1.

**
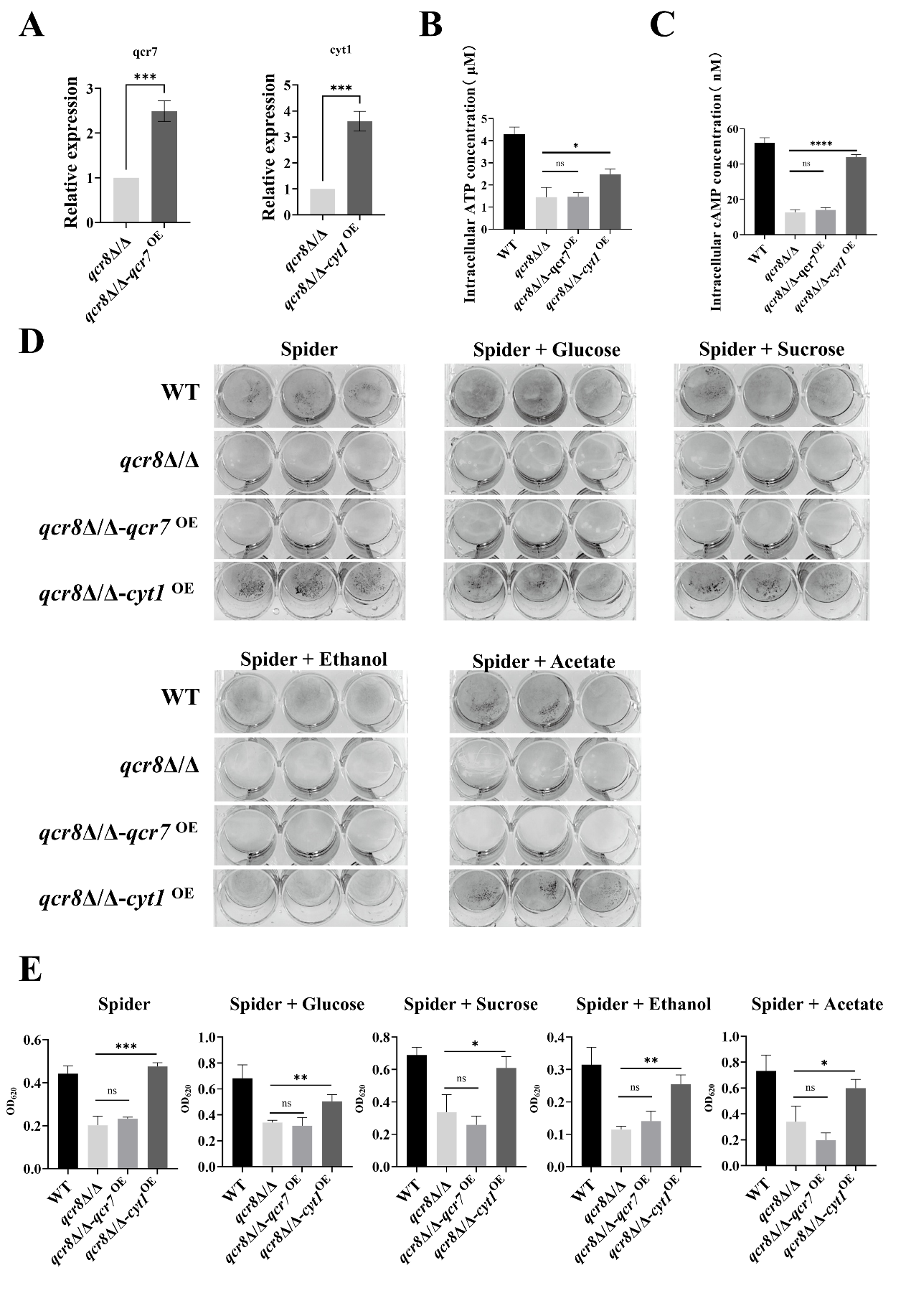
**

**Figure S10 Phenotypic characterization of *qcr8*Δ/Δ strains overexpressing *qcr7* and *cyt1.*** (A) RT-qPCR analysis showing the relative expression levels of *qcr7* and *cyt1* in *qcr8*Δ/Δ-*qcr7* ^OE^ and *qcr8*Δ/Δ-*cyt1* ^OE^ strains compared with *qcr8*Δ/Δ.
(B) Intracellular ATP concentrations of WT, *qcr8*Δ/Δ, *qcr8*Δ/Δ-*qcr7* ^OE^, and *qcr8*Δ/Δ-*cyt1* ^OE^ strains.
(C) Intracellular cAMP concentrations of WT, *qcr8*Δ/Δ, *qcr8*Δ/Δ-*qcr7* ^OE^, and *qcr8*Δ/Δ-*cyt1* ^OE^ strains.
 (D) Adhesion assay of WT, *qcr8*Δ/Δ, *qcr8*Δ/Δ-*qcr7* ^OE^, and *qcr8*Δ/Δ-*cyt1* ^OE^ strains grown in Spider medium or Spider medium supplemented with glucose, sucrose, ethanol, or acetate.

(E) Quantification of adhesion (OD600) of WT, *qcr8*Δ/Δ, *qcr8*Δ/Δ-*qcr7* ^OE^, and *qcr8*Δ/Δ-*cyt1* ^OE^ strains under the same conditions as in panel D.

**Table S1. The changes of four prediction models**

| **Interaction Type** | **Full Complex** | **Missing QCR7** | **Missing QCR8** | **Missing QCR7 + QCR8** |
| --- | --- | --- | --- | --- |
| **Hydrogen bonds** | 31 | 30 | 11 | 34 |
| **Salt bridges** | 4 | 3 | 3 | 3 |
| **π-Stacking** | 1 | 1 | 1 | 1 |
| **π-Cation interactions** | 2 | 1 | 0 | 2 |
| **Functional impact** | Stable | Minor Impact | Major Impact | Partial Restoration |

**Table S2. Primers used in this study**

| Primer name | Sequence (5’ to 3’) |
| --- | --- |
| Leu left | CATGCAGAACCAGAACTCAATG |
| Leu right | CTCACTTGATTCTGATTGGCTC |
| His left | CAGTCTCTTGAAGTTTTCGATCG |
| His right | CATGAGCACCATAAGGACGTTC |
| Arg left | CGAAGGTCACACTGACTTATGTC |
| Arg right | GACGTGTGGTAATATTGGTTGA |
| up F | CTACATTAACTGGTATACTCCGG |
| up R | CACGGCGCGCCTAGCAGCGGGTGGGTGGGTTTGTAAGATTG |
| down F | GTCAGCGGCCGCATCCCTGCGCAACAATTGATTCTTCATAATCATG |
| down R | GGCATTAGCAATGTATATGAGTGG |
| up check F | GACTGTCCGATAACACAAGAGTT |
| down check R | GTTGAAGGAGTTAACAACATTGC |
| Target check F | AGGTGCACCACATCCACATAC |
| Target check R | CTTCTCTACCTTCTTTAGTGTACAAGTATTC |
| Primer 5 | CCGCTGCTAGGCGCGCCGTGAGCTCGGATCCACTAGTAACG |
| Primer 2 | GCAGGGATGCGGCCGCTGACGCCAGTGTGATGGATATCTGC |
| 18S-F | CGCAAGGCTGAAACTTAAAGG |
| 18S-R | AGCAGACAAATCACTCCACC |
| GAPDH-F | ACCACAGTCCATGCCATCAC |
| GAPDH-R | TCCACCACCCTGTTGCTGTA |
| Ras1-F | ATCAAGATGGATTAGCATTGG |
| Ras1-R | TGTTGTTGCTGTTGTTGTTG |
| Cyr1-F | \| AGAAAGAAGACGATGAAACAG \| \| --- \| |
| Cyr1-R | AGGAGAACTAGAGGATGTAGAC |
| Tpk1-F | CAAGGTGGTTCTGATGATGTG |
| Tpk1-R | ACGATATGGGTCTTCAACTCC |
| Tpk2-F | AACAACCGCAGCAACAACTT |
| Tpk2-R | GAATGGAGTGATGAGGTAGCAGAT |
| Efg1-F | CAGTGGTGGCAGTAATGTGTCT |
| Efg1-R | CAGTGGTGGCAGTAATGTGTCT |
| Flo8-F | CAGCAGCAGCAACAACAACAA |
| Flo8-R | CACTAGCAACACCGATTCCTCTT |
